# Effect of Pulsed Electric Fields and Osmotic Dehydration on the Quality of Modified-Atmosphere-Packaged Fresh-Cut and Fried Potatoes

**DOI:** 10.3390/foods14030420

**Published:** 2025-01-27

**Authors:** Efimia Dermesonlouoglou, George Seretis, Maria Katsouli, Alexandros Katsimichas, Petros Taoukis, Maria Giannakourou

**Affiliations:** Laboratory of Food Chemistry and Technology, School of Chemical Engineering, National Technical University of Athens (NTUA), 9, Iroon Polytechniou Str, 15772 Zografou, Greece; gseretis123@gmail.com (G.S.); mkatsouli@chemeng.ntua.gr (M.K.); alkats@chemeng.ntua.gr (A.K.); taoukis@chemeng.ntua.gr (P.T.)

**Keywords:** *Solanum tuberosum*, osmotic dehydration, pulsed electric fields, browning, texture, quality, deep drying, storage

## Abstract

The aim of this research was to study the effect of osmotic dehydration (OD) and/or pulsed electric field (PEF) on the quality of MAP-packed potatoes, both as raw materials and after deep frying. Fresh-cut potato strips (from Naxos island) were osmotically dehydrated using a solution of 20% glycerol, 5% sodium chloride, and 1% ascorbic acid (wt) at a 5:1 liquid-to-food ratio at 35 °C for 120 min. OD-treated and untreated samples were packaged at MAP (0.2% O_2_ + 12% CO_2_) and stored at 4, 8, and 12 °C. Color (Browning Index, *BI*), texture (hardness, *F_max_*), sensory characteristics (including total sensory quality), and microbial stability (total aerobic and anaerobic counts, *Pseudomonas*, Entrobacteriaceae, and yeasts/molds) were monitored during storage. After package opening, samples were deep-fried at 180 °C for up to 8 min, and the oil content of fried samples was quantified. Sensory evaluation of raw and fried samples was conducted. Untreated fresh-cut potatoes were characterized by detrimental color degradation starting from the third day of storage at 4 °C and presented microbial growth (total viable counts: 6 log (CFU)/g) on the sixth day, whereas pre-treated potato samples retained their color and microbiological stability after 15 and 18 days of cold storage, respectively. OD pre-treatment reduced the oil uptake during frying (up to 30%).

## 1. Introduction

Potatoes are highly popular due to their rich content of nutrients, minerals, and dietary fibers and their versatility in various food applications, such as potato fries [1,2,3]. However, during the fresh-cutting process, the mechanical damage to the tissue makes them susceptible to enzymatic browning and reduces their shelf life [4,5,6]. The browning of fresh-cut fruits and vegetables occurs when phenolic compounds undergo enzymatic oxidation, catalyzed by polyphenol oxidase, leading to the formation of quinones, which then condense into colored pigments [4,5]. Blanching (at 55–75 °C for a minimum of 10 min) is commonly used in the production of par-fried potatoes to deactivate polyphenol oxidase. However, blanching requires significant amounts of water and is not energy efficient [7]. Additionally, during blanching, a colorless complex may form between potato iron and chlorogenic acid. Upon oxidation, this complex can create ferri-chlorogenic acid, resulting in an undesirable dark color. To mitigate this, anti-browning agents, such as sulfites, are often added to the blanching solution [8]. Previous studies have also highlighted the positive effect of osmotic dehydration (as a single treatment or in combination with other technologies) in preserving the color (browning) of fresh-cut fruits and vegetables [9,10,11,12,13,14,15,16].

Osmotic dehydration (OD) is a process where food is immersed in a hypertonic solution—containing carbohydrates, salts, and other specific ingredients such as antioxidants and antimicrobials—at mild temperatures. The key parameters of this process include temperature, time, food-to-osmotic solution ratio, and the osmotic solution formulation [9,10]. For each food product, the optimal processing conditions must be chosen to ensure efficient dehydration within a reasonable timeframe while preserving food quality. The influence of different parameters, particularly temperature, time duration, and the composition of the osmotic solution, on mass transfer during OD has been widely studied [10,12]. Osmotic dehydration has been extensively explored as a pre-treatment method, particularly before drying or freezing foods with a high water content. Studies have shown that OD pre-treatment can increase the quality and stability of fresh-cut fruits and vegetables [9,10,13,14,15,16,17]. Additionally, the impact of OD on frying kinetics and the quality of potato fries (French type) has been explored by Krokida et al. [18], Gupta et al. [19], and Kwaw et al. [20]. The studies found that pre-frying dehydration reduced oil absorption in potato fries while significantly enhancing their color and structural properties. Osmotic dehydration also helped prevent discoloration that occurred during subsequent frying.

Recently, there has been increasing interest in innovative technologies that offer significant potential for reducing energy consumption and shortening processing times [16,21,22]. Among these, pulsed electric field (PEF) has emerged as a promising process for various food applications owing to its low power requirements [16,21,22]. PEF is recognized as an effective pre-treatment for enhancing mass transfer processes, such as dehydration (air drying, freeze drying, and osmotic dehydration) and extraction [23,24,25,26,27]. The process involves the use of high-voltage pulses on semi-liquid or liquid foods placed between two electrodes. This electric field induces electroporation, which creates pores in the cell membranes and walls. As a result, cell permeability increases, allowing intracellular water to move into the extracellular matrix [23,28]. This movement of water accelerates dehydration but also leads to a loss of turgor pressure, causing tissue softening [23,24,29,30]. PEF technology is now used in the potato industry to reduce cutting forces [30,31]. Research has also explored the potential of PEF pre-treatment on potato tubers for French fries production [30,31,32,33,34,35,36,37]. PEF treatment has been shown to enhance the permeability of potato cell membranes, reduce oil and acrylamide content in fried potatoes, and improve their texture [30,36,37]. However, studies on the combined use of PEF (as a pre-treatment for osmotic dehydration) and osmotic dehydration before frying are still relatively limited.

The objective of this study is twofold: (1) to assess and model the impact of pulsed electric field (PEF) pre-treatment conditions on the osmotic dehydration (OD) parameters (including mass transfer) and product quality characteristics (water activity, texture, and color) of fresh-cut potatoes and (2) to evaluate the quality and shelf-life stability (with regard to microbial growth) of potatoes treated with OD and PEF + OD and packaged under modified atmosphere during cold storage. The quality of OD-treated and PEF + OD-treated potatoes during/after frying was also determined (oil uptake during frying, texture/color, and sensory properties of the fried product). For this study, potatoes with protected geographical indication certifications from Naxos Island (Greece) were used. Preserving the unique qualities of “Naxos” potatoes at their freshest while extending their shelf life is essential from a quality standpoint. This will ensure that they can be successfully distributed to both domestic and foreign markets.

## 2. Materials and Methods

### 2.1. Materials

Potatoes (of the Spunta variety) were purchased from a local potato producer (SKLIRAKIS, J.,-JACOB GIAKOUMIS O.E., Naxos, Greece) at the maturity stage (based on crop physiology and the presence of a thick, firm skin) and stored at room temperature in the dark for 7 days (maximum). The potatoes with similar color, size, and maturity and no obvious disease, insect, and mechanical damage were peeled (1–2 mm) and cut along the longitudinal direction (in the middle of the potato) to obtain potato strips of weight 5 g and length 5 cm. The water content of the raw material was 0.874 ± 0.024 g water/g sample weight.

### 2.2. Pulsed Electric Field (PEF) Treatment of Potatoes

Potato strips were treated in water (tap, with an electrical conductivity of 800 μS/cm) at an electric field strength of 0.5 kV/cm for 200 pulses, resulting in a specific energy input equal to 0.02 kJ/kg [38]. The treatment was performed in a stainless-steel parallel plate batch electrode chamber with an electrode spacing of 8 cm and a total volume of 300 mL. Bipolar pulses of near-rectangular shape were delivered to the treatment chamber using an Elcrack-5 kW unit (DIL, Quackenbrück, Germany). In brief, approximately 80 g of potato strips were transferred to the treatment chamber for each PEF treatment and covered with tap water. The pulse width was set to 15 μs, the amplitude of electric pulses was 4 kV, and the pulse frequency was maintained at 20 Hz. The initial temperature of potato strips was approximately 25 °C, and the post-treatment temperature remained practically unchanged. Sample temperature was measured by a digital thermometer (General Tools & Instruments, Secaucus, NJ, USA) before and after each PEF treatment. After each treatment, selected quality parameters (texture/color) of the potatoes were evaluated.

### 2.3. Osmotic Dehydration (OD) of Untreated and PEF-Pre-Treated Potatoes

Potato strips (untreated and PEF-pre-treated) were first weighed and placed in cylindrical glass containers, which were then filled with preheated osmotic medium of 20% glycerol, 5% sodium chloride, and 1% ascorbic acid at 35 °C to achieve a food-to-liquid sample ratio of 1:5. The containers were immersed in a thermostatic water bath with continuous agitation at 240 rpm (Grant GL5400 Linear Shaking Water Bath, Royston, UK). Samples were taken at 0, 30, 60, 120, and 180 min, rinsed with water to remove excess solution, and gently blotted with absorbent paper [32]. Water loss (*WL*), solid gain (*SG*), water activity (*a_w_*), and the quality properties of texture (firmness) and color (browning) change were determined throughout the OD process.

Osmotic dehydration of potatoes for the shelf-life study was performed in pilot-scale equipment consisting of a cylindrical (Ø28 × 60 cm) stainless steel 100 L tank fitted with spigots, which allowed draining of the solution. Stirring was also mildly applied (set at a rotational speed of 120 rpm) through an electrical motor paddle-type stirrer, carefully submerged into the solution. A heating option was also provided, where required, with a complementary stainless steel helical tube, which could be heated via a continuous circulation of water from a 3 kW water bath. In our work, gentle heating was employed (temperature set at 35 °C, monitored with a thermocouple throughout the whole process) to ensure homogeneous dissolution of the osmotic medium constituents. The solution required for osmotic dehydration was prepared in situ within the stainless-steel tank by dosing the ingredients using a high-capacity electronic balance. Potatoes were manually loaded and unloaded in the tank (batch process). After OD treatment, the potatoes were rinsed with centrifugal household vegetable equipment and placed for 30 min on a perforated rack to drain off excess osmotic solution. The scaling-up of the OD process up to the 100 kg scale was deemed successful, and the measured parameters agreed with their counterparts obtained after experiments in the lab scale.

### 2.4. Quality Monitoring of Untreated and PEF-Pre-Treated OD Potatoes During Cold Storage

OD potato strips, untreated and PEF-treated, were packaged in PA (polyamide)/PE (polyethylene) bags (250 × 175 × 0.04 mm; ten potato strips from each repetition) in modified atmosphere (12.2% CO_2_-0.188% O_2_) (Boss NT42N, Bad Homburg, Germany) and stored in controlled temperature cabinets at 4, 8, and 12 °C (±0.2 °C) (Sanyo MIR 153, Sanyo Electric, Osaka, Japan). The temperature in the cabinets was continuously monitored using temperature data loggers (COX TRACER, Belmont, NC, USA). At predetermined sampling storage times, three bags per processing with one bag per repetition were selected for the shelf-life study. Quality (texture/color) and microbial stability of samples were measured. Gas headspace of packages was analyzed (CheckMate 9900 O_2_/CO_2_, PBI Dansensor, Ringsted, Denmark).

OD potato strips, untreated and PEF-treated, were fried in sunflower oil at 180 °C (±1 °C) for 0, 2, 4, 6, 8, and 10 min (Tefal Visialis Deep Fryer, Groupe SEB, Paris, France). The fryer was filled with 2 L of oil in a potato strips-to-oil ratio of 1:50 *w*/*v*. Fried potato strips were drained for a few seconds on paper.

### 2.5. Analytical Protocols and Mathematical Modeling

#### 2.5.1. Physicochemical Parameters

Water activity (*a_w_*) was determined by a water activity meter (Aqua lab 4TEV, Decagon Devices, Pullman, WA, USA). The water (moisture) content was measured by drying at 105 °C for 18 h (WTB BINDER 7200, Type C53, Tuttlingen, Germany) [39]. pH was measured using a pH meter (338, Amel Instruments, Milano, Italy). Ten grams of homogenized potato sample was diluted in 90 mL of sterilized Ringer solution [40].

##### Mass Transfer During Osmotic Dehydration

Water loss (*WL*) and solid gain (*SG*) values were calculated using Equations (1) and (2) [41], respectively.

(1) $$WL=\frac{m_{0}-m_{0}\cdot DW_{wb}-\left(m_{wet}-m_{dry}\right)}{m_{0}\cdot DW_{wb}}$$

(2) $$SG=\frac{m_{dry}-DW_{wb}\cdot m_{0}}{DW_{wb}\cdot m_{0}}$$

where *m*_0_ represents the initial wet weight of the sample prior to immersion in the osmotic solution, *DW_wb_* is the dry weight of the untreated sample (g of water/g of sample wet base), *m_wet_* is the wet weight of the treated sample (g), and *m_dry_* is the dry weight of the OD-pre-treated sample (g).

According to our kinetic approach, the penetration model was used to describe water loss and solid gain, where the ratio of WL/SG is proportional to the square root of the contact time, according to Equation (3) [41].

(3) $$WL\;or\;SG=k_{X}\cdot \sqrt{t_{OD}}$$

where *WL* is in $\frac{g\;water}{g\;DW}$ or *SG* is in $\frac{g\;solid}{g\;DW}$ at time *t_OD_*; *k_X_* is the water loss constant (*k_WL_*) in g waterg DW·s12 or the solid gain constant (*k_SG_*) in g solidsg DW·s12; and *t* is the osmotic dehydration time in min.

#### 2.5.2. Potato Product Composition

The glycerol content of the potato strip product was measured according to the assay proposed by Kuhn et al. [42] with minor modifications. The final measurement was executed in a microplate reader at 410 nm over a period of 25 min. The final glycerol content was calculated based on a glycerol standard curve and expressed as mg glycerol/g potato. The salt content of the potato strip product was measured titrimetrically by the Mohr method [39]. Vitamin C (mg L (ascorbic acid)/100 g potato) of the potato strip product was determined by a high-performance liquid chromatography method [43]. For all measurements, two replicates were used.

#### 2.5.3. Potato Product Firmness

Potato strips were compressed to a deformation of 20% by a knife attached to a texture analyzer (TA-XT2i, Stable Micro Systems, Godalming, UK) (test speed of 1.00 mm/s; distance of 4.00 mm). The knife was used to cut the potatoes along the longitudinal direction in the middle of each potato sample. The firmness was recorded as the maximum compression force (*F_max_*, N) [11]. A minimum of five replicates were performed for each measurement.

#### 2.5.4. Potato Product Color

An Xrite-i1 portable digital colorimeter (Gretag-Macbeth, Grand Rapids, MI, USA) was used to assess the objective color on the potato surface, expressed in the CIE-Lab color scale. At least five replicates were taken by measuring the color at three different evenly distributed locations on the surface of each potato. The degree of browning was quantified using the Browning Index (*BI*) calculated using Equation (4) [44]. In the present study, *BI* is used (as the most representative parameter) to indicate color changes in the processed potato strips during OD processing as well as during cold storage [1,2,3,45].

(4) $$BI=\frac{100\left(\frac{\left(a\;+\;1.75\;\times \;L\right)}{\left(5.645\;\times \;L\;+\;a\;-\;0.3012\;\times \;b\right)}-0.31\right)}{0.17}$$

where *L*, *a*, and *b* represent the measured CIE-Lab color parameters.

#### 2.5.5. Oil Content

Five grams of smashed potato strips were weighed and placed in a 250 mL beaker together with hexane at a 1:10 sample–solvent ratio. The mixture was allowed to stir with a magnetic stirrer for about 40 min, and the liquid solvent–oil phase was then placed in pre-weighed 100 mL round-bottom flasks by filtration. The vacuum evaporator (Heidolph G1, Germany) was then used to evaporate the hexane at a temperature of 40 °C, with gentle rotation of the flask. Finally, the spherical bottles were re-weighed, and the gross bottle-oil weight was measured, with the fat content calculated through the weight and the initial amount.

For the prediction of the oil uptake during the frying process, a first-order kinetic model proposed in the literature was used [32] (Equation (5)).

(5) $$Y=Y_{e}[1-\exp\left(-k_{OIL}\times t_{fr}\right)]$$

where *Y* is the oil content (kg/kg db), *Y_e_* is the equilibrium value of *Y* (kg/kg db), *k_OIL_* is the rate constant (min^−1^), and *t_f_*_r_ is the frying time.

#### 2.5.6. Sensory Analysis

The sensory evaluation was performed by eight to ten trained adult assessors [46]. Participation in the assessments was voluntary, and an informed written consent was obtained by all participants prior to the sensory evaluation study, which could be withdrawn at any point of the evaluation. The organizer of the sensory test provided a detailed description of the test to all participants to inform them about the food samples that would be assessed. A questionnaire was also distributed where they were obliged to report any indispositions or allergies, and if such was the case, the particular volunteer was excluded from the tests.

Sensory attributes of fried potato strips (including the appearance and odor of the raw samples) were assessed, with scores recorded on appropriate forms to reflect the organoleptic changes indicating quality deterioration (provided as a Supplementary Materials). The fried potato strips were described in terms of appearance (oil detection and color browning), odor, texture (crispiness, firmness, and gumminess), taste (overall, sweet taste, salty taste, and other), flavor, aftertaste, and total sensory quality. Each sensory parameter, as well as the total sensory quality, was scored separately on a 1–9 scale (where 9 represented high quality and 1 represented poor quality). A sensory score of 5 was considered the threshold for minimum acceptability.

#### 2.5.7. Microbial Growth

The growth of key microorganisms associated with the spoilage of fresh-cut potatoes was monitored throughout storage. For microbiological analysis, a 10 g sample was transferred to a sterile stomacher bag containing 90 mL of sterilized Ringer solution (Merck, Darmstadt, Germany) and homogenized for 60 s using a Stomacher (Bag Mixer, Interscience, Saint Nom la Bretêche, France). Mold and yeast counts were performed using Rose Bengal Chloramphenicol (RBC, Merck, Darmstadt, Germany) after incubation at 25 °C for 120 h. Total aerobic mesophiles were counted using the standard plate count method with plate count agar (PCA, Merck, Darmstadt, Germany) incubated at 25 °C for 72 h. Total anaerobic bacteria were enumerated on sulfite-polymyxinsulfadiazin-A (SPS) agar, with plates incubated at 35 ± 0.5 °C for 24–36 h in anaerobic jars with an atmosphere generation system (Oxoid, Basingstoke, Hampshire, UK). *Pseudomonas* spp. was measured on cetrimide agar (CFC, Merck, Darmstadt, Germany) after incubation at 25 °C for 48 h. The pour plate method was used for Enterobacteriaceae enumeration using violet red bile glucose agar (VRBG, Merck, Darmstadt, Germany) after incubation at 37 °C for 24–48 h. Two replicates from at least three appropriate dilutions were enumerated. Microbial counts were expressed as log CFU per gram of tissue. Microbial growth was modeled using the Baranyi growth model. The DMFit program (available at www.combase.cc accessed on 24 July 2024) was used for curve fitting and calculation of the kinetic parameters, lag phase (*λ* in d), and microbial growth rate (*k_micr_* in d^−1^).

### 2.6. Statistical Analysis

Analyses were conducted on at least three duplicate samples, and treatments were conducted at least twice. The mean value ± SD is used to report the results. The significance level for analyses of variance (ANOVA) was set at *p* < 0.05 using Statista 7.0 (StatSoft, Inc., Tulsa, OK, USA). As a post hoc analysis, Duncan’s multiple range test was used to separate means with significant differences.

## 3. Results and Discussion

### 3.1. PEF and OD

#### 3.1.1. Effect of PEF and OD on Mass Transfer and Water Activity of Potatoes

**Figure 1:**
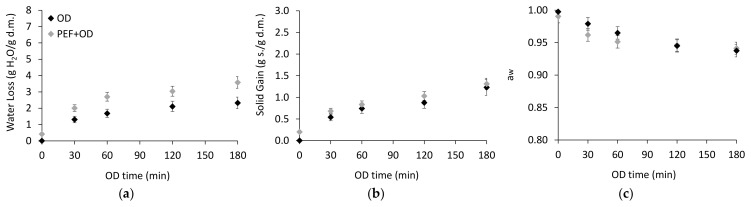
Mass transfer: (**a**) water loss (*WL*, g water/g d.m.), (**b**) solid gain (*SG*, g s./g d.m.), and (**c**) water activity (*a_w_*) during osmotic dehydration (OD) of OD-treated and PEF + OD-treated potato strips. (values: mean ± standard error).

For both untreated and PEF-pre-treated potato strips, water loss increased and water activity rapidly decreased at the start of the OD process, reaching stable values after 120 min, as shown in Figure 1a,c. PEF pre-treatment significantly affected water loss up to 180 min of OD (*p* < 0.05). The increase in water loss after PEF processing was also noted by the increase in the water loss constant (*k_WL_*) (Equation (3); Table 1). The effect of PEF on solid gain values (under the applied processing conditions) was not significant (*p* > 0.05) (*k_SG_*) (Figure 1b; Table 1). Dimopoulos et al. (2024) reported that PEF pre-treatment of osmotically dehydrated spinach (glycerol concentration in the osmotic solution: 60%) led to a significant *WL* as well as *SG* increase [41]. Dermesonlouoglou et al. (2019) reported that the increase in glycerol concentration (from 40 to 60%) in the osmotic solution significantly affected both the water and the solids diffusion of OD peach fruit [47]. In this study, the glycerol concentration was kept low (at 20%), aiming at a minimum alteration of potato sensory attributes.

Pre-treatment of OD potatoes with PEF induced slight changes in the product water activity (*a_w_,_PEF/OD sample_*: 0.9375; *a_w_,_OD sample_*: 0.9406) (*p* > 0.05). Significantly different *a_w_* values for OD-treated and PEF + OD-treated potato strips were observed for osmotic dehydration times up to 60 min (*p* < 0.05). According to Katsouli et al. (2024) [38], although the mass transfer and water activity values are significantly lower at more intense process conditions (PEF application as a pre-osmotic dehydration step), the temporal evolution (rate of *a_w_* decrease) is not affected by PEF treatments. This observation suggests that electroporation leads to an immediate release of free water right after the treatment. A rapid immersion in the osmotic solution is adequate to eliminate this released free water and reduce water activity.

#### 3.1.2. PEF and OD Effect on Color and Texture of Potato Strips

**Figure 2:**
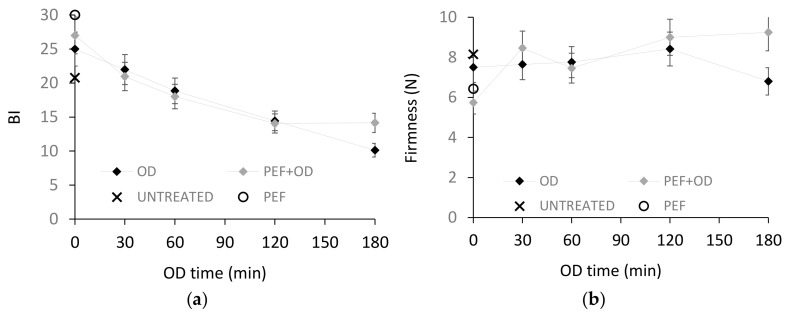
(**a**) Browning index (*BI*) and (**b**) firmness (*F_max_*, N) values of raw (untreated), OD-treated, PEF-treated, and PEF + OD-treated potato strips during osmotic dehydration (0, 30, 60, 120, and 180 min) (values: mean ± standard error).

PEF pre-treatment influenced the quality of the product, specifically in terms of color degradation and texture firmness during osmotic dehydration (OD). The degradation of color in treated potatoes as a function of the duration of osmotic dehydration was quantified by the Browning Index (*BI*) (Figure 2a). The *BI* values for samples subjected to PEF pre-treatment were found to be higher compared to those of the OD-treated samples. Furthermore, the application of OD to PEF-treated samples resulted in a decrease in *BI* values. Throughout the OD process, *BI* values for both standard OD-treated and PEF + OD-treated samples exhibited a decline. It was observed that after 120 min of OD, the *BI* values approached 14.0. For OD times > 120 min and *BI* values < 14, the potato strips were characterized as «white-colorless». Katsouli et al. (2024) [38] similarly noted that the *BI* values of potato strips subjected to PEF treatment prior to osmotic dehydration (OD) were lower than those of untreated samples. The firmness of potatoes post-PEF treatment is a significant aspect, as it affects the sensory attributes of the final product. Figure 2b illustrates the variation in firmness of the samples in relation to the duration of osmotic dehydration. A softening effect was observed in the potatoes following both PEF and OD treatments, with firmness decreasing from approximately 8.15 N in the fresh-cut samples to 6.43 N and 5.74 N after PEF and PEF + OD treatments, respectively. This finding is consistent with the observations made by Katsouli et al. (2024) [38] regarding potato strips treated with PEF and OD. Conversely, during the osmotic dehydration process, an increase in firmness values was recorded. Specifically, a 120 min duration of OD resulted in firmness values that approached those of fresh-cut potatoes, i.e., approximately 9.00 N. The OD-treated and PEF + OD-treated potato product characteristics for OD time of 120 min (selected as the appropriate OD treatment time) are shown in Table 2. The PEF + OD-treated potato strips were characterized by significantly lower water activity and pH values compared to the values of the untreated potato strips. PEF + OD-treated potato strips presented different color and texture parameters. The OD-treated potato strips were positively evaluated during the sensory evaluation testing. Regarding the composition of the OD-modified and PEF + OD-modified potato tissue, glycerol, salt, and ascorbic acid (vitamin C) content were calculated by analytical methods.

### 3.2. Quality of OD-Treated and PEF + OD-Treated Potatoes After Frying

#### 3.2.1. Oil Uptake During Frying of OD-Treated and PEF + OD-Treated Potato Strips

**Figure 3:**
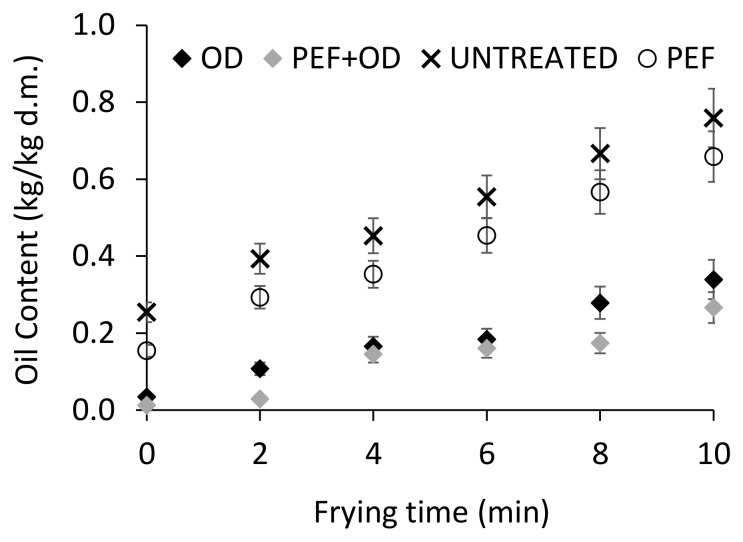
Oil content (kg/kg d.m.) during frying (values: mean ± standard error).

The oil content of potatoes during frying up to 10 min is presented in Figure 3. OD, as well as the combined PEF and OD application, significantly decreased the oil uptake during frying (*p* < 0.05). For all sample categories, the data for oil uptake showed an exponential increase in oil uptake with the increase in frying time. In Table 3, the oil content constants for different pre-treatments (OD, PEF, PEF + OD) are reported as calculated by Equation (5) after appropriate data fitting.

Pre-drying procedures lower the crust’s permeability, moisture content, and oil absorption of fried products. When potatoes are dried, a skin layer forms on their surface, which lowers porosity and lessens the amount of vapor that passes through the surface. There is less vacant space for oil to remain after frying when porosity decreases. Because the dry matter level of potatoes contributes to the undesirable mealy texture of fries, manufacturers try to increase the dry matter of potatoes by pre-drying them before frying. Chips made from tubers with a high dry matter content absorb less oil when fried. Oladejo et al. (2017) investigated the impact of osmotic dehydration on the quality attributes of French fries [48]. They found that samples that were pre-treated in osmotic solutions had much less oil and moisture compared to the untreated samples [48]. They reported that the increased moisture movement inside the food product’s structure during frying, which results in high vapor pressure within the food structure and reduces oil absorption, could be the cause of the reduced oil content of the osmotically pre-treated samples. Furthermore, a crust formed on the surface of the fried samples treated with OD, which may have stopped the oil from being absorbed during the cooling process. This outcome matches the findings of Krokida et al. (2001) [32], Karizaki et al. (2013) [33], Dehghannya et al. (2015) [49], and Barani et al. (2020) [50]. Krokida et al. (2001) reported that the oil content reduced as drying time increased [32].

The positive effects of electroporation were also highlighted by Liu et al. (2017, 2018) [51,52], who observed a reduction in oil absorption during the frying of potato discs. Their research found that PEF treatment was more effective than a blanching-induced gel layer in reducing oil uptake in French fries. Specifically, PEF-treated chips showed a 38.7% reduction in oil uptake, while blanching only resulted in a 3.8% decrease [51]. PEF treatment reduces oil absorption by promoting water diffusion from the potato core to the surface, which forms a thicker water vapor layer that minimizes both dehydration and oil absorption during frying. Additionally, the smoother surface of PEF-treated potatoes enhances oil drainage after frying. Regardless of the processing conditions, PEF treatments increase the moisture content and softens the potato cubes without altering their fresh appearance. More recently, it has been shown that combining PEF treatment with convective air drying significantly shortens frying times and reduces oil absorption in fried potatoes [52]. Furthermore, the efficiency of potato dehydration was notably improved when PEF treatment was combined with vacuum drying at sub-atmospheric pressure [53].

#### 3.2.2. Quality of OD-Treated and PEF + OD-Treated Fried Potato Strips

**Figure 4:**
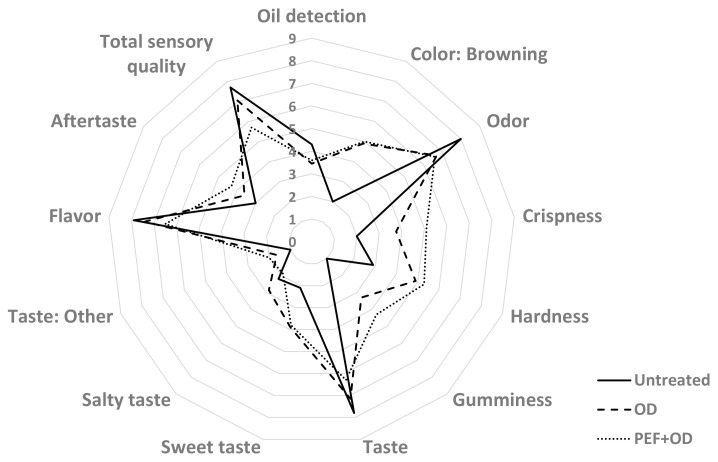
Average scores (scale 1–9) for sensory characteristics of untreated (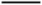), OD-treated (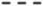) and PEF + OD-treated (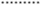) fried potato strips (180 °C, 10 min): oil detection, color browning, odor, crispiness, firmness, gumminess, taste overall, sweet taste, salty taste, other taste, flavor, aftertaste, and total sensory quality.

The organoleptic quality of fried potato strips (frying time 10 min) can be described in terms of appearance (oil detection and color browning), odor, texture (crispiness, firmness, and gumminess), taste (overall, sweet taste, salty taste, and other), flavor, aftertaste, and total sensory quality. In Figure 4, average scores (scale 1–9) for the characteristics (axis 1–13) of untreated, OD-treated, and PEF + OD-treated potato strips are demonstrated.

Fresh-cut potatoes and/or minimally processed potatoes are convenient but highly perishable products. Unlike most fresh-cut vegetables, which are ready to eat, fresh-cut potatoes must be cooked before consumption. Therefore, in addition to the safety (chemical and microbiological), quality, and sensory characteristics of raw fresh-cut potatoes, the same requirements should be applied for cooked potatoes [54]. In the present study, the sensory evaluation was conducted on raw potatoes as well as fried potatoes. Sensory scores for the individual sensory/quality parameters as well as the overall sensory quality of these samples were presented and mathematically modeled. Score for overall sensory quality encompasses the panelists’ general opinion about the examined samples and is a good indicator of the evolution of a product’s quality.

From the sensory scores, the OD-treated fried potato strips received a high score for the total sensory quality (7.1/9.0 compared to 7.7/9 for the untreated fried potato sample). PEF pre-treatment negatively affected the sensory quality of the OD-treated fried samples. PEF + OD-treated fried potato strips received the lowest score for total sensory quality (6.0/9.0), mainly due to their increased firmness. PEF-pre-treated potato strips presented the value of 10 N for firmness compared to 8 N of the OD-treated sample and 5.5 N of the untreated sample. The water content of untreated, OD-treated, and PEF + OD-treated samples was calculated as 0.413, 0.249, and 0.298 g/g, respectively. All treated samples had a sticky texture due to glycerol content compared to the untreated sample.

French fries have two distinct textures: a “crispy” crust, similar in physical characteristics to potato chips (or crisps) [55], and a “firm-mealy” core, which shares some of the textural properties of boiled potatoes. The key structural parameters that influence the crispness of French fries include moisture content, oil absorption, and the starch content and distribution within the potato tissues. Other important parameters affecting crispness are related to the manufacturing process, such as pre-drying and par-frying conditions [56,57].

PEF-treated samples received lower scores for aroma, taste, and oil content. Both OD-treated and PEF + OD-treated samples were accepted in terms of appearance and presented improved sensory characteristics. Previous studies of Abedpour and Dehghannya (2016) [58] and da Costa Ribeiro et al. (2016) [59] on osmotic dehydration pre-treatment of potato preceding frying revealed that OD pre-treatment improved sensory characteristics of the fried potato.

**Scheme 1:**
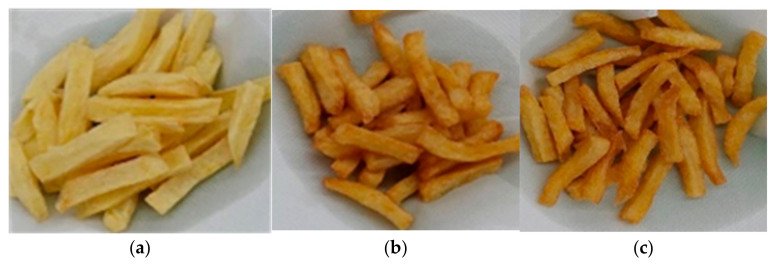
Photos of (**a**) untreated, (**b**) OD-treated, and (**c**) PEF + OD-treated potato strips after deep frying at 180 °C for 10 min (day 0 of the storage).

The OD-treated and PEF + OD-treated samples exhibited a dark yellow color, in contrast to the light yellow color of the untreated sample (Scheme 1). Among these, the OD-treated samples were considered the most visually appealing. The golden yellow hue of the fried crust is an important factor in consumer preference, often influencing their choice even before tasting. This color results from the Maillard reaction, which leads to the caramelization of sugars when fried at high temperatures [37,59]. The intensity of the color is influenced by the presence of reducing sugars, amino acids, and proteins on the surface, as well as the frying temperature, edible coating, and pre-treatment method [19,28,60]. Zhang et al. (2018) reported that combining PEF treatment with blanching significantly enhanced the brightness of French fries, giving them a golden yellow appearance [61]. The discoloration observed during frying was primarily caused by the Maillard reaction, with the depth of color depending on the levels of reducing sugars, amino acids, or proteins on the surface of the potato strips, as well as frying temperature and duration [60]. Blanching likely resulted in the loss of reducing sugars and amino acids due to electrolyte leakage from the potato strips. As a result, the PEF–blanching combined pre-treatment slowed the Maillard reaction and significantly reduced browning during frying [61,62].

### 3.3. Shelf-Life Study of OD and PEF + OD Raw Potatoes

#### 3.3.1. Texture and Color Change During Storage

**Figure 5:**
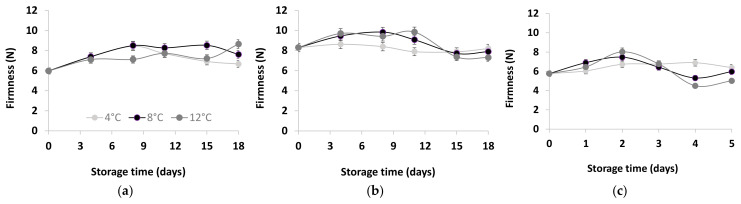
Evolution of texture (firmness, N) of (**a**) OD-treated, (**b**) PEF + OD-treated, and (**c**) untreated potato strips during their storage at 4, 8, and 12 °C.

The texture (firmness) and color (browning) of OD raw potato strips were retained during 18 days of storage. In Figure 5, the firmness change of OD-treated, PEF + OD-treated, and untreated potato strips during storage are presented. PEF-treated samples presented the highest firmness values compared to the untreated and OD-treated samples. During the 18 days of storage, the firmness of OD-treated and PEF + OD-treated samples increased, whereas the firmness of untreated samples remained stable during their shorter 5 days of storage. The firmness of OD-treated samples presented a 10%, 22%, and 31% increase at the end of their storage at 4, 8, and 12 °C, respectively. The firmness values of PEF + OD-treated samples significantly decreased at a storage temperature of 12 °C (12%). Firmness in potato tubers is influenced by three primary changes that occur due to chemical, physical, and structural transformations during the manufacturing process [63]. First, starch undergoes gelatinization; second, cell walls weaken, resulting in increased permeability; and third, the adhesion between adjacent cells diminishes [64]. The extent of these changes is determined by thermal conditions, including processing temperature and treatment duration.

**Figure 6:**
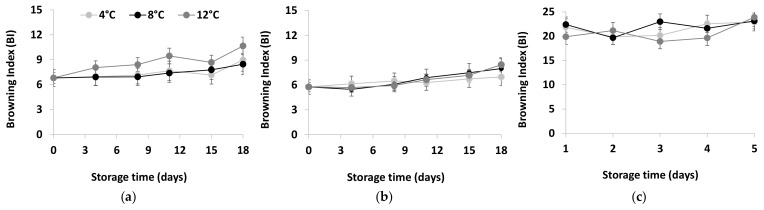
Evolution of color (Browning Index, *BI*) of (**a**) OD-treated, (**b**) PEF + OD-treated, and (**c**) untreated potato strips during their storage at 4, 8, and 12 °C.

Browning index values were significantly lower for OD-treated (66%) and PEF + OD-treated (71%) potato strips (at day 1 and throughout storage) compared to untreated ones, showing that OD pre-treatment protected potato color, which is a major problem of fresh-cut potatoes (*p* < 0.05). In Figure 6, the *BI* changes of OD-treated, PEF + OD-treated, and untreated potato strips during storage are presented. The *BI* values of untreated samples were 20.48 (day 1) and 23.89 (day 5 at 12 °C) compared to *BI* values of OD-treated and PEF + OD-treated samples, which were 6.81 and 5.76 (day 1) and 10.66 and 8.45 (day 5 at 12 °C), respectively. *BI* values of all samples slightly increased during storage. The same trend was observed by Katsouli et al. [38] for PEF + OD-treated and high-pressure (HP) + OD-treated potato strips, which presented lower *BI* values (and lighter color) compared to untreated potato strips. More specifically, HP and PEF reduced the *BI* by 12.5% and 27.9%, respectively, compared to the samples that had only been osmo-dehydrated.

The potato variety Spunta, commonly cultivated for the fresh market in southern Europe, the Middle East, and North Africa, is among the most susceptible to browning [65]. Therefore, for such delicate tissue, the milder color (PEF and OD) could be considered commercially significant. However, despite the stability of the *BI* value of the untreated sample during the 5 days of storage, untreated samples were rejected at day 3 by the sensory panelists. This could be due to the discolorations on the surface of the potato strips. Although color measurements were taken at three representative points of the potato tissue, the *BI* failed to accurately reflect the color differences. Therefore, visual inspection proves to be a more reliable method for monitoring processing. In a study on enzymatic browning in potatoes, Cantos et al. (2002) assessed color development using both the *BI* and organoleptic evaluation given the unique characteristics of the tissue and observed discrepancies between the two methods [65].

#### 3.3.2. Evolution of Microbial Load During Storage

**Figure 7:**
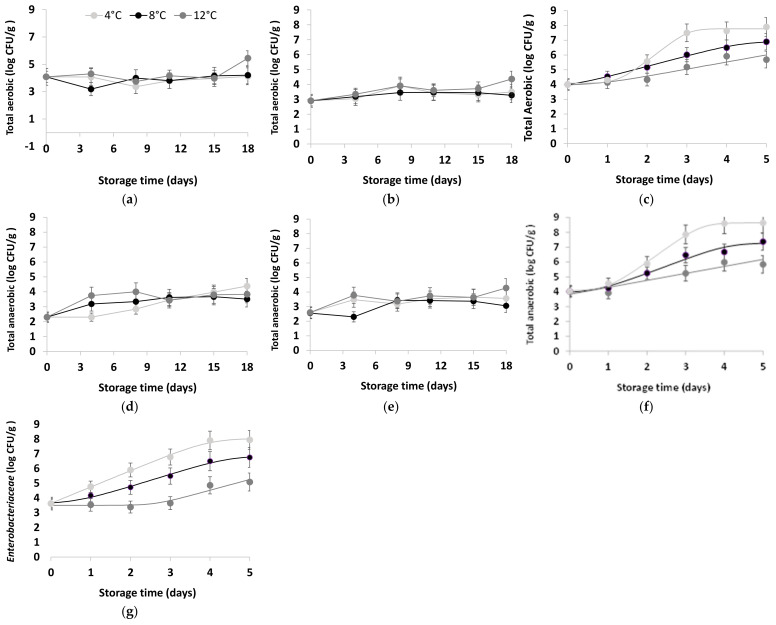
Microbial growth: total viable counts (TVC) of (**a**) OD-treated, (**b**) PEF + OD-treated, and (**c**) untreated samples; total anaerobic count of (**d**) OD-treated, (**e**) PEF + OD-treated, and (**f**) untreated samples; and (**g**) Enterobacteriaceae of untreated potato strips. (**a**,**d**,**g**) Experimental values and (**a**–**c**) experimental values and data fit to the Baranyi model. Error bars represent the standard deviation of three replicates.

In the present study, the primary focus was on the quality changes incurred by spoilage, microbial growth, and/or enzymatic activity. The evolution of the total aerobic bacteria, Enterobacteriaceae, and anaerobic microorganisms (no significant growth of other microorganisms was observed) of untreated, OD-treated, and PEF + OD-treated potato strips is shown in Figure 7. OD pre-treatment significantly increased the microbial stability of potato strips. The microbial counts of OD-treated and PEF + OD-treated potatoes presented an extended lag period before reaching about 10^4^–10^5^ CFU/g at 18 days of storage, as also reported by Dimopoulos et al. (2024) [41]. The microbial counts of untreated potatoes reached 10^5^ (4 °C)–10^8^ (12 °C) CFU/g (total anaerobic) after 5 days of storage. The initial total viable load (TVC) of untreated potato strips was 4 log(CFU)/g. The total viable counts for pre-treated with OD and PEF + OD were <6 log(CFU)/g for 18 days of chill storage. Enterobacteriaceae were at levels below the limit of detection (<10 CFU/g). In the case of the untreated sample, Enterobacteriaceae growth was observed after 5 days of storage. The kinetic parameters of the microbial growth model, microbial growth rate (*k*), and lag phase (*λ*) are presented in Table 4. The effect of OD treatment regarding microbial growth inhibition can be attributed to the difficulty of microorganism penetration into cellular spaces, which are filled with the concentrated osmotic solution [66]. Glycerol, chosen as the primary agent for reducing water activity in the osmotic medium, also demonstrates benefits as a microbial protectant. The combination of water activity levels, pH values, and packaging conditions in the developed osmo-dehydrated potatoes ensures their microbial stability.

#### 3.3.3. Evolution of Sensory Quality Loss During Storage

The quality of fresh-cut potatoes (slices, strips, or cubes) is defined by their bright appearance, firm texture, slightly moist surface, and flesh color characteristic of the variety, free from signs of darkening or dehydration. To preserve these qualities, a combination of anti-browning agents, such as sodium bisulfite or organic acids, is commonly used alongside modified atmosphere packaging or vacuum packaging to maintain very low oxygen levels. Key parameters influencing quality include the potato cultivar, harvesting and handling practices, tuber maturity, and the peeling method employed. In the present study, the modified atmosphere of 12.2% CO_2_–87.6% N_2_ (0.188% O_2_) used for the package kept the microbial counts at low levels for 5 and 18 days of storage at 4 °C (untreated, OD-treated, and PEF + OD-treated samples, respectively). During storage, the O_2_ and CO_2_ concentration levels decreased for all temperatures studied. For example, the O_2_ and CO_2_ concentrations of untreated potatoes stored at 4 °C reduced from 0.188 and 12.2 to 0.139 and 9.0, respectively. The same was observed for OD-treated and PEF + OD-treated samples, to a lesser extent. The respective values for OD-treated and PEF + OD-treated samples were 0.122 and 10.9 (OD) and 0.121 and 11.4 (PEF + OD). Ma et al. (2010) demonstrated that potato slices could be preserved for 8 days at 5 °C under a controlled atmosphere of 12% CO_2_ and 3% O_2_, combined with a dip in 0.025% sodium bisulfite [67]. Similarly, a shelf life exceeding 7 days at 5 °C was achieved when bisulfite was replaced with citric and ascorbic acids and packed under a modified atmosphere of 20% CO_2_ and 80% N_2_ [68]. Angos et al. (2008) reported that a super-atmospheric oxygen composition of 80% O_2_ and 10–20% CO_2_ effectively controlled browning and suppressed the respiration rate compared to a controlled atmosphere with 2.5% O2 and 10% CO2 over 14 days of storage at 4 °C [69]. Additionally, an active modified atmosphere with initial O_2_ levels of 0.5–1.0% using a 35 μm thick polypropylene (PP) film preserved the appearance of fresh-cut potatoes for 10 days at 4 °C, though slight off-odors developed [70]. Modified atmosphere packaging (MAP) with low oxygen levels (1–3%) at 0–5 °C can reduce cut surface browning but is insufficient to completely prevent it [71]. A shelf life of up to 3 weeks at 2 °C can be achieved by applying an active MAP with an initial nitrogen flush, resulting in a gas composition of 2–5% O_2_ and 3–5% CO_2_ during storage [72]. These gas levels do not impact microbial populations compared to storage in air [73]. Beltran et al. (2005) concluded that vacuum packaging was the most effective method for preserving the sensory quality of fresh-cut potatoes for up to 14 days at 4 °C. Under MAP conditions, browning was inhibited only with sodium sulfite, but this treatment led to the development of off-odors after 14 days at 4 °C [74].

In the present study, the OD-treated potato samples were rejected on day 15 due to off-odor development. The untreated fried potato samples were rejected on day 3 due to discoloration (based on sensory deterioration as well as microbial growth). As reported, (sole) MAP packaging could not maintain the color of fresh-cut potatoes [74]. The OD-treated and PEF + OD-treated fried potato strips were sensorially evaluated up to day 15 for the three temperatures studied. The average sensory scores for OD-treated potato fries were 6.5, 6.3, and 6.0 for the storage temperatures of 4, 8, and 12 °C, respectively, showing that the effect of temperature was not statistically significant. The same was observed for PEF + OD-treated samples. PEF + OD-treated samples received 6.5 (4 °C), 6.4 (8 °C), and 6.3 (12 °C) on storage day 15. The shelf life of OD-treated as well as PEF + OD-treated fried potato strips was limited to 15 days at 4–12 °C by the sensory rejection of (raw, after package opening) potato strips. Both OD-treated and PEF + OD-treated potato samples were microbially stable, as shown in Section 3.3.2.

## 4. Conclusions

The scope of this study was to explore the application of PEF and OD for effective treatment of potatoes with the aim of retaining or improving the quality and shelf life of modified-atmosphere-packaged raw potato strips as well as reducing oil uptake during subsequent frying. OD (35 °C, 120 min) with/without PEF (0.5 kV/cm, 200 pulses) pre-treatment was found to promote the retention of the overall quality (texture and color) and increase the shelf-life stability of the fresh-cut potato strips. Additionally, the OD-treated and PEF + OD-treated samples resulted in high-quality potato strips with reduced browning during chilled storage. Raw OD-pre-treated potatoes were microbiologically stable for 18 days at 4 °C; however, their shelf life was estimated at 15 days based on sensory acceptance. The untreated potato strips were rejected at 3 days (based on sensory deterioration as well as microbial growth). OD pre-treatment reduced the oil uptake during frying (up to 30%) and resulted in potato fries judged positively by sensory panelists. OD in combination with MAP can extend the shelf life and improve the commercial value of fresh-cut potatoes (especially concerning the “Naxos” variety, which has a special added value), while OD pre-treatment can be utilized as an alternative method of producing low-fat potato fries.

A point to consider regarding PEF and OD feasibility in the food industry is related to existing technical and economic challenges and scalability but also to the need for optimization of these emerging technologies. Taking also into account each technology’s limitations, their successful combined use may assist in delivering safer and environmentally friendly fresh-cut potato products with prolonged shelf life. More specifically, OD, while retaining sensory qualities, cannot provide the required microbial stability and consumes large volumes of osmotic solution, increasing operational costs and rendering scalability difficult. On the other hand, PEF, although energy efficient, is not yet fully explored regarding the optimization of processing parameters, and validation of results on a larger scale remains a point of investigation. Nonetheless, PEF has been extensively explored and recently implemented in various fresh-cut product treatments, which makes it a promising technology for broader commercial application in the fresh-cut potato industry. A strong competitive advantage of PEF is that it has already been used in the potato industry. In this context, future research should focus on addressing energy efficiency issues for smaller-scale operations and designing an appropriate optimization study to ensure consistency and scalability, especially in terms of texture and sensory quality. Future studies should also investigate the effect of these technologies on different potato cultivars and processing conditions to retain the initial superior quality of raw material and maximize the shelf life of the end product [16].

## Figures and Tables

**Table 1:** Water loss (*WL*) and solid gain (*SG*) constants for osmotic dehydration and/or pulsed electric fields (OD and PEF + OD) (Equation (3)).

	Water Loss Constant kWLg waterg DW·s12	R^2^	Solid Gain Constant kSGg solidsg DW·s12	R^2^
OD-treated	0.1913 ± 0.0095 ^a^	0.9885	0.0893 ± 0.0088 ^a^	0.9948
PEF + OD-treated	0.2320 ± 0.0151 ^b^	0.9708	0.0798 ± 0.0068 ^a^	0.9927

Different superscript letters indicate significant differences between means *k*_WL_ and *k*_SG_ ± standard deviation as calculated by Duncan’s multiple range test for a significance level of *p* = 0.05.

**Table 2:** Potato product characteristics (untreated, OD-treated, and PEF + OD-treated).

Sample	Water Content	*a_w_*	pH	Vitamin C (g asc.acid/g)	Glycerol (g /g)	Salt (g NaCl/g)	Color (*L, a, b, BI*)	Texture (Firmness, *F_max_*, N)	Sensory Score (1–9)
Untreated	0.8572	0.9927	6.036	0.0013	_	0.004	53.77; −0.57; 20.48	5.76	7.9
OD-treated	0.7014	0.9462	4.798	0.011	0.372	0.054	54.22; −0.98; 8.10	8.41	7.1
PEF + OD-treated	0.6821	0.9457	4.468	0.010	0.356	0.049	52.99; −1.17; 7.88	9.55	5.7

**Table 3:** Oil uptake constant (*k*_*OIL*_) values for untreated, OD-treated, PEF-treated, and PEF + OD-treated potato strips as calculated by Equation (5) after fitting to the experimental data.

	Oil Uptake Constant *k*_*OIL*_ $\left({min}^{-1}\right)$	R^2^
Untreated	0.199 ^a^	0.9483
PEF-treated	0.198 ^a^	0.9875
OD-treated	0.162 ^b^	0.9302
PEF + OD-treated	0.142 ^b^	0.8961

Different superscript letters indicate significant differences between means *k_OIL_* ± standard deviation as calculated by Duncan’s multiple range test for a significance level of *p* = 0.05.

**Table 4:** Baranyi model parameters for microbial growth (total viable counts (TVC), Enterobacteriaceae, and total anaerobic count) for untreated potato strips. The dependence of the rate constant *μ_m_* on storage temperature was modeled with the Arrhenius equation.

Storage Temperature (°C)	$\mu_{m}(d^{-1})$	$logN_{o}\left(\frac{logCFU}{g}\right)$	$logN_{o}\left(\frac{logCFU}{g}\right)$	R^2^	$E_{a,\mu m}\left(\frac{kJ}{mol}\right)$	$\mu_{m},ref(d^{-1})$	R^2^
Total Viable Count (TVC)							
4	0.483 ± 0.14	3.977 ± 0.318	0.801 ± 1.159	0.839	118.96	0.839	0.9317
8	0.718 ± 0.053	3.998 ± 0.083	0.293 ± 0.219	0.995			
12	2.062 ± 0.326	4.085 ± 0.142	1.267 ± 0.201	0.990			
Enterobacteriaceae							
4	0.734 ± 0.206	3.508 ± 0.157	2.611 ± 0.559	0.876	33.61	0.335	0.9513
8	0.834 ± 0.105	3.670 ± 0.156	0.668 ± 0.362	0.984			
12	1.106 ± 0.058	3.643 ± 0.111	_	0.994			
Total Anaerobic Count							
4	0.523 ± 0.153	3.758 ± 0.309	_	0.858	104.88	0.518	0.9995
8	0.975 ± 0.243	3.972 ± 0.319	0.703 ± 0.640	0.944			
12	1.876 ± 0.145	4.084 ± 0.122	0.982 ± 0.147	0.996			

## Data Availability

The data presented in this study are available on request from the corresponding author. The data are not publicly available due to project grant confidentiality restrictions.

## Supplementary Materials

The following supporting information can be downloaded at https://www.mdpi.com/article/10.3390/foods14030420/s1, Table S1: Tested sensory characteristics for raw and fried potatoes.
