# Supplementary material for: Effect of Pulsed Electric Fields and Osmotic Dehydration on the Quality of Modified-Atmosphere-Packaged Fresh-Cut and Fried Potatoes
Source: Foods. 2025 Jan 27;14(3):420. doi: 10.3390/foods14030420 (PMC11816707; doi:10.3390/foods14030420)
Supplement: Supplementary file 1 [file foods-14-00420-s001.zip › foods-3412006-supplementary.pdf]

Table S1. Tested sensory characteristics for raw and fried potatoes.

Characteristic	Adopted scale
**Characteristics pertaining to raw potatoes**	
Glycerol detection	Intensity scale 1-9
Intensity of product irregularities in the packaging	Intensity scale 1-9
Intensity of color/browning	Intensity scale 1-9
Intensity of color/other (dark spots)	Intensity scale 1-9
Product odor liking	Hedonic scale 1-9
Intensity of odor/other	Intensity scale 1-9
Intensity of perceived crispness	Intensity scale 1-9
Intensity of perceived hardness	Intensity scale 1-9
Product texture liking	Hedonic scale 1-9
**Characteristics pertaining to fried potatoes**	
Oil detection	Intensity scale 1-9
Intensity of color/browning	Intensity scale 1-9
Product odor liking	Hedonic scale 1-9
Intensity of perceived crispness	Intensity scale 1-9
Intensity of perceived hardness	Intensity scale 1-9
Intensity of perceived gumminess	Intensity scale 1-9
Product taste liking	Hedonic scale 1-9
Intensity of sweet taste	Intensity scale 1-9
Intensity of salty taste	Intensity scale 1-9
Intensity of other taste	Intensity scale 1-9
Product flavor liking	Hedonic scale 1-9
Intensity of aftertaste	Intensity scale 1-9
Total sensory quality/liking	Hedonic scale 1-9
